# Bridging the Diagnostic Gap: Enhancing Orthopedic Infection Management through Laboratory Diagnostics in Nepal

**DOI:** 10.31729/jnma.v63i292.9246

**Published:** 2025-12-31

**Authors:** Ram Bahadur Khadka, Samrat Parajuli, Nabin Pahari

**Affiliations:** 1 Department of Medical Laboratory Science, Crimson College of Technology, Pokhara University, Butwal, Rupandehi, Nepal; 2 Department of Orthopedic Surgery, Lumbini Hospital and Technical College, Butwal, Rupandehi, Nepal; 3 Department of Emergency Medicine, Lumbini Provincial Hospital, Butwal, Rupandehi, Nepal

**Keywords:** *bone diseases*, *clinical laboratory technique*, *orthopedics*, *Nepal*

## Abstract

Orthopedic infections, including implant-associated infections, periprosthetic joint infections, and osteoarticular tuberculosis, pose significant diagnostic and therapeutic challenges globally. In Nepal, limited laboratory facilities outside major cities often lead to empirical treatment, resulting in delayed diagnosis, inappropriate use of antibiotics and rising antimicrobial resistance. This viewpoint indicates the importance of integrating microbiological diagnostics into orthopedic practice in resource limited settings. It highlights key laboratory tools, outlines the barriers to their effective use, and proposes locally adaptable strategies to improve diagnostic accuracy, treatment outcomes, and antibiotic stewardship in orthopedic infection care.

## INTRODUCTION

Orthopedic infections, including surgical site infections (SSI), periprosthetic joint infections (PJI), osteomyelitis, septic arthritis, open fracture-related infections and osteoarticular tuberculosis represent major health challenges globally and in Nepal. Periprosthetic joint infections occurs in 1-2.5% of primary and upto 4% of revision surgeries.^1^ Surgical site infections account for 10-20% of healthcare associated infections worldwide.^2^ Infections rates in open fracture reach upto 19%.^3^ Osteoarticular tuberculosis forms 10% of extrapulmonary cases.^4^ In Nepal surgical site infection reach 31.2% with 47.7% culture positivity.^5-9^ Limited diagnostic drive empirical therapy and resistance.^10-12^ This highlights the need to strength laboratory support, based on global and local evidence.

## ROLE OF LABORATORY

Laboratory investigations are central to the diagnosis, management and monitoring of orthopedic infections in Nepal. Microbiological culture of pus, synovial fluid or periprosthetic tissue remains the gold standard for pathogen identification, enabling targeted therapy, though its yield may be reduced by prior antibiotic exposure or improper specimen handling.^13,15^ Antibiotic susceptibility testing (AST) is crucial for guiding therapy; however, access remains inconsistent in regional hospitals with delays,cost, and limited capacity affecting timely interventions.^14,16^

Basic systemic inflammatory markers like as complete blood count (CBC), erythrocyte sedimentation rate (ESR) and C-reactive protein (CRP) are widely available and routinely used, even in secondary level hospitals, to support diagnosis and monitor treatment.^13,14^ Advanced biomarkers like procalcitonin (PCT) are confined to tertiary or private facilities due to high costs and are used mainly in severe or uncertain cases.^16^

In tuberculosis endemic regions, GeneXpert MTB/RIF allows rapid detection of Mycobactrium tuberculosis and rifampicin resistance, though availability is limited to provincial or referral hospitals.^17,18^ Smaller centers still rely heavily on conventional AFB Stains or hisopathoglogy.^17^ Histopathological examination is particularly important for confirming osteomyelitis, skeletal tuberculosis, tumors or granulomatous lesions, especially when microbiological results are inconclusive , though delayed reporting is a challenge.^15,17^

Advanced molecular diagnostics, including PCR, nucleic acid amplification tests, and 16s rRNA gene sequencing hold promise for detecting fastidious or biofilm associated organisms, but their use is largely confined to research or specialized laboratories in Kathmandu and Pokhara.^19,20^

**Table 1:** Common Laboratroy Tests In Orthopedic Care

Test	Availability in Nepal	Cost (NPR)	Clinical Utility	Limitations	Solutions/Local Adaptation
Gram Stain and Culture	Widely available in most hospitals	400-600	Confirms diagnosis in osteomyelitis, septic arthritis, implant infections	Time consuming, false negatives if prior antibiotics	Obtain samples before antibiotics; use enriched media; larger hospitals more likely to perform.^13,14^
Antibiotic Susceptibility Test (AST)	Available in tertiary/Private labs; limited in regional hospitals	500-1000	Guides targeted antibiotic therapy	Limited in smaller labs; turnaround 48-72 hrs	Use in tertiary/private labs; complement with clinical judgment.^14,16^
ESR	Widely available in most hospitals	100-200	Useful for diagnosis and monitoring chronic bone/joint infections	Non-specific; elevated in anemia, malignancy	Combine with CRP and clinical correlation.^13,14^
CRP	Widely available in most hospitals	300-500	Monitors infection and treatment response	Non-specific	Serial measurement; widely available even in regional labs.^14^
Procalcitonin (PCT)	Limited availability; high cost;rarely used in routine orthopedic care	2500-4800	Helps to differentiate bacterial from viral/non-infectious causes	High cost;limited availability	Use selectively in severe or uncertain cases; mainly tertiary centers.^16^
AFB Stain/GeneXpert MTB/RIF	GeneXpert available in selected tertiary and provincial hospital; routine use in bone and joint specimens limited	4400-4800	Essenstial for skeletal TB diagnosis	GeneXpert limited outside tertiary labs; low sensitivity in bone	Combine with biopsy, histopathology; expand molecular platforms gradually.^17,18^
Histopathology	Available in most hospitals; delayed reporting in resource-poor labs	1000-2000	Confirms osteomyelitis, TB, tumors	Invasive; delayed reporting	Upgrade lab capacity; coordinate with microbiology.^15,17^
Serum Calcium, Phosphate, Alkaline Phosphatese	Available in tertiary/private labs; limited in regional hospitals	300-500	Assesses metabolic bone disease	Non-specific for infection	Combine with radiology and clinical assessment.^14^
Blood Culture	Available in tertiary/private labs; limited in regional hospital	500–1000	Critical in hematogenous osteomyelitis	May be false negative; contamination risk	Repeat culture; mainly in tertiary/private labs.^13,15^
PCR/16s rRNA Sequencing	Mostly restricted to research or specialized labs in Kathmandu/Pokhara; not yet widely accessible in regional centers	5000–10000	Detects fastidious organism	Requires specialized equipment; limited availability	Use in research/specialized labs; plan regional lab expansion.^19,20^

Overall, laboratory investigations ranging from basic inflammatory markers to advanced molecular tools are pivotal for improving diagnostic accuracy, guiding rational antibiotic use and combating antimicrobial resistance in orthopedic infection in Nepal.^13-20^ A summary of commonly used laboratory tests in this context are shown in Table 1.

## CHALLENGES

In Nepal, utilization of laboratory support in orthopedic care faces multiple challenges. Studies from tertiary hospitals indicate that suboptimal sample collection, like as superficial swabs instead of deep aspirates, reduces culture sensitivity.^13,15^ Laboratory infrastructure limitations are notable with molecular platforms like PCR and GeneXpert being largely restricted to research or select tertiary centers.^17,19^ Diagnostic delays are common when advanced testing, including histopathology or next-generation sequencing must be referred to external laboratories.^15,17^ Evidence regarding pre-emptive antibiotic use prior to culture collection in Nepali hospitals remains limited; no formal audit or survey data from regional hospitals currently quantify this practice.^14,16^ Finally, the lack of standardized diagnostic protocols and interdepartmental coordination has been identified in hospital reports as a barrier to consistent care.^15,17^ These findings highlights the need for structured local audits and prospective studies to generate concrete data on laboratory utilization and clinical practices in Nepal.

**Figure 1:**
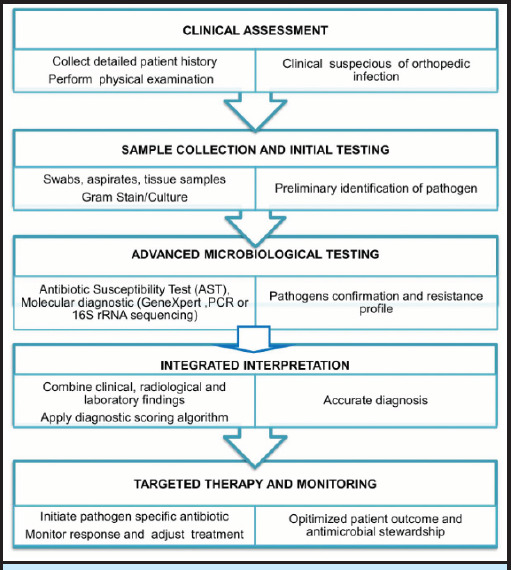
A simplified stepwise diagnostic strengthening strategy for orthopedic infection in Nepal.

## WAY FORWARD

Improving orthopedic infection diagnostics in Nepal and other low-resource settings requires practical, actionable strategies that directly guide clinicians toward timely diagnosis and treatment. A stepwise approach can begin with clinical assessment and basic inflammatory markers followed by targeted specimen collection for Gram stain and culture before initiating antibiotics, with AST results used to guide therapy. In suspected osteoarticular tuberculosis, GeneXpert MTB/RIF or histopathology can support diagnosis, while advanced molecular tests such as PCR or 16S rRNA sequencing can be centralized in referral centers and applied selectively for recurrent or complex cases (Figure 1). Standardized protocols for sample collection, processing and reporting together with interdisciplinary collaboration among orthopedic surgeons, microbiologist, and infectious diseases specialists are essential. Progress can be monitored through measurable indicators such as culture positivity rates, AST utilization, reduction in empirical antibiotic use, and time to initiation of targeted therapy. Implementing a simple scoring system or algorithm incorporating clinical, laboratory, and radiological findings could further standardize diagnosis and treatment, thereby improving patient outcomes and promoting effective antimicrobial stewardship.

## LIMITATIONS

This viewpoint is derived from a synthesis of available peer-reviewed literature, relevant national and international guidelines, and the authors clinical and laboratory experience in Nepal. However, it does not incorporate national surveillance or multicenter data, and some conclusions are based on limited published evidence and expert interpretation. Therefore, the findings and recommendations should be considered indicative rather than comprehensive, underscoring the need for broader data collection and prospective studies to validate and expand these insights.

## References

[1] Tande AJ, Patel R (2014). Prosthetic Joint Infection.. Clin Microbiol Rev.

[2] Mengistu DA, Alemu A, Abdukadir AA, Husen A, Ahmed F, Mohammed B (2023). Global Incidence of Surgical Site Infection Among Patients: Systematic Review and Meta-Analysis.. INQUIRY.

[3] Swiontkowski M, Cross W (2008). Treatment Principles in the Management of Open Fractures.. Indian J Orthop.

[4] Garg RK, Somvanshi DS (2011). Spinal Tuberculosis: A Review.. The Journal of Spinal Cord Medicine.

[5] Basnet A, Joshi P, Shrestha SKD, Khanal LK, Karmacharya M, Shrestha S (2024). Antimicrobial Resistance in Bacterial Species Causing Orthopaedic Surgical Site Infections at a National Trauma Center, Kathmandu, Nepal.. The American Journal of Tropical Medicine and Hygiene.

[6] Pradhan I, Regmi S, Kunwar M, Basukala B, Joshi A (2022). Positive Bacterial Culture Among Suspected Orthopedic Infections in a Tertiary Care Centre: A Descriptive Cross-Sectional Study.. J Nepal Med Assoc.

[7] Shrestha RC, Sharma GR, Bhattachan M, Aryal S (2020). Outcome of Micro-Lumbar Discectomy and Preventive Measures to Control Discitis.. Nep J Neurosci.

[8] Adhikari S, Paudel S, Kafle D, Pokharel RK (2020). Incidence and Risk Factors of Surgical Site Infection in Prolapsed Lumbar Intervertebral Disc Surgery.. Nepal Orthop Assoc J..

[9] Das M, Pandey S, Das A (2025). Rate of Infection in Open Fracture of Long Bones with Delayed Debridement.. J Coll Med Sci-Nepal.

[10] Stevenson MC, Slater JC, Sagi HC, Palacio Bedoya F, Fletcher MV (2022). Diagnosing Fracture-Related Infections: Where Are We Now?. J Clin Microbiol..

[11] Mahato RK, Htike KM, Koro AB, Somlorm K, Yadav RK, Kafle A (2025). Geographical Patterns of Tuberculosis Notification Rates and Their Association With Socioeconomic Factors in Nepal: A Spatial Cross-Sectional Study (2020-2023).. BMJ Open..

[12] Agrawal P, Giri B, Gupta P, Nepali A, Parajuli S (2024). Utilization of Diagnostic Services at a Municipal Hospital in Rural Nepal: Perspective from a General Practitioner-Led Primary Care Delivery.. jgpeman.

[13] Jha B, Gautam S, Sharma J, Sharma M (2021). Bacteriological Profile and Antimicrobial Resistance Pattern in Surgical Site Infection in a Tertiary Care Hospital, Central Nepal.. Med. J. Shree Birendra Hosp..

[14] Bhatta DR, Adhikari A, Gurung JL, Amatya NM, Nayak N, Gokhale S (2021). Bacteriological Profile of Surgical Site Infections in a Tertiary Care Hospital of Western Nepal.. J. Gandaki Med. Coll. Nepal.

[15] Baral R, Sherpa P, Gautam D, KC SR (2020). Histopathology of Extra-Pulmonary Tuberculosis at Pathology Lab of Patan Hospital, Nepal.. J Patan Acad Health Sci.

[16] Chan YL, Tseng CP, Tsay PK, Chang SS, Chiu TF, Chen JC (2004). Procalcitonin as a Marker of Bacterial Infection in the Emergency Department: An Observational Study.. Crit Care..

[17] Chaudhary R, Bhatta S, Singh A, Pradhan M, Shrivastava B, Singh YI (2021). Diagnostic Performance of Genexpert Mtb/rif Assay Compared to Conventional Mycobacterium Tuberculosis Culture for Diagnosis of Pulmonary and Extrapulmonary Tuberculosis, Nepal.. Narra J.

[18] Gurp M, Rood E, Fatima R, Joshi P, Verma SC, Khan AH (2020). Finding Gaps in Tb Notifications: Spatial Analysis of Geographical Patterns of Tb Notifications, Associations with Tb Program Efforts and Social Determinants of Tb Risk in Bangladesh, Nepal and Pakistan.. BMC Infect Dis.

[19] Bio Diagnostics Lab. Lab Tests..

[20] National Public Health Laboratory. Patient Portal..

